# Lysosomal processing of progranulin

**DOI:** 10.1186/s13024-017-0205-9

**Published:** 2017-08-23

**Authors:** Xiaolai Zhou, Daniel H. Paushter, Tuancheng Feng, Lirong Sun, Thomas Reinheckel, Fenghua Hu

**Affiliations:** 1 Department of Molecular Biology and Genetics, Weill Institute for Cell and Molecular Biology, Cornell University, 345 Weill Hall, Ithaca, NY 14853 USA; 2 Department of Neurobiology, School of Basic Medical Sciences, Southern Medical University, Guangzhou, China; 3 Institute of Molecular Medicine and Cell Research, Medical Faculty and BIOSS Centre for Biological Signalling Studies, Albert-Ludwigs-University Freiburg, 79104 Freiburg, Germany

**Keywords:** Progranulin (PGRN), Cathepsin, Lysosome, Frontotemporal lobar degeneration (FTLD), Neuronal ceroid lipofuscinosis (NCL)

## Abstract

**Background:**

Mutations resulting in progranulin (PGRN) haploinsufficiency cause frontotemporal lobar degeneration with TDP-43-positive inclusions (FTLD-TDP), a devastating neurodegenerative disease. PGRN is localized to the lysosome and important for proper lysosome function. However, the metabolism of PGRN in the lysosome is still unclear.

**Results:**

Here, we report that PGRN is processed into ~10 kDa peptides intracellularly in multiple cell types and tissues and this processing is dependent on lysosomal activities. PGRN endocytosed from the extracellular space is also processed in a similar manner. We further demonstrated that multiple cathepsins are involved in PGRN processing and cathepsin L cleaves PGRN in vitro.

**Conclusions:**

Our data support that PGRN is processed in the lysosome through the actions of cathepsins.

## Background

Progranulin (PGRN) is an evolutionarily conserved glycoprotein of 7.5 granulin repeats encoded by the granulin (*GRN*) gene in humans [1–4]. Mutations in the *GRN* gene are associated with several neurodegenerative diseases [1–4]. While PGRN haploinsufficiency is a leading cause of frontotemporal lobar degeneration (FTLD) [5], complete loss of PGRN is known to cause neuronal ceroid lipofuscinosis (NCL) [6, 7], a group of lysosomal storage diseases. Accumulating evidence suggests an important function of PGRN in the lysosome. Transcription of the *GRN* gene is regulated by the transcriptional factor, TFEB, together with a number of essential lysosomal genes [8], and PGRN is trafficked to lysosomes through two distinct pathways [9, 10]. However, the metabolism of PGRN in the lysosome remains to be determined. One interesting hypothesis is that PGRN is processed into granulin peptides in a similar manner to prosaposin (PSAP), the precursor of saposin peptides (A, B, C, D) that are essential for lysosomal glycosphingolipid metabolism [11–13], and that granulins function to regulate enzymatic activities in the lysosome [2].

## Results

### Intracellular processing of PGRN

**Fig. 1:**
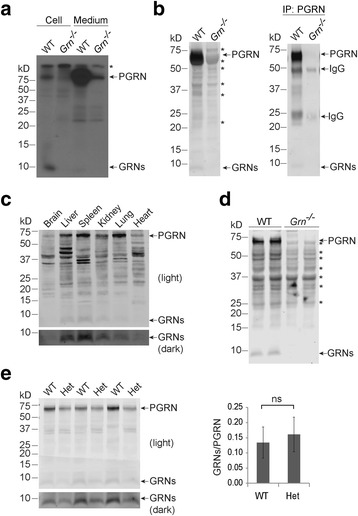
Intracellular processing of PGRN. **a** Primary microglia from WT and *Grn* ^*−/−*^ mice were labeled with [^35^S]methionine and [^35^S]cysteine for 24 h. Cell lysates and media were immunoprecipitated by homemade rabbit anti-PGRN antibodies and separated by 16% Tricine-SDS PAGE. The PGRN and PGRN-derived peptide (GRNs) signals were visualized by autoradiography. * indicates non-specific bands. Please note there is a weak non-specific band that is the same size as full-length PGRN in both lysate and medium. **b** PGRN processing in MEF cells. Equal amounts of cell lysate from primary WT and *Grn* ^*−/−*^ MEF cells (*left*) and the rabbit anti-PGRN IP products from WT and *Grn* ^*−/−*^ MEF cells (*right*) were separated on 4–12% Bis-Tris gels and blotted with sheep anti-mouse PGRN antibodies (1:1000). * indicates non-specific bands. Please note that PGRN runs slightly differently on Tricine gels and Bis-Tris gels. **c** PGRN processing in mouse tissues. Equal amounts of tissue lysates were separated on a 4–12% Bis-Tris gel and blotted with sheep anti-mouse PGRN antibodies (1:1000). **d** Brain tissue from WT and *Grn* ^*−/−*^ adult mice were lysed with RIPA buffer at a ratio of 1:10 (g:ml) and an equal amount of protein was separated on a 4–12% Bis-Tris gel and immunoblotted with sheep anti-mouse PGRN antibodies (1:300). **e** Spleen tissues from WT and *Grn* ^*+/−*^ (Het) adult mice were lysed with RIPA buffer at a ratio of 1:10 (g:ml) and an equal amount of protein was separated on a 4–12% Bis-Tris gel and immunoblotted with sheep anti-mouse PGRN antibodies (1:1000). The ratios between granulin peptides (GRNs) and PGRN were quantified. ns: not significant, student’s T-test

To test the potential processing of PGRN, we immunoprecipitated PGRN and any potential PGRN-derived peptides from primary microglia grown in [^35^S]-labeled methionine- and cysteine-containing medium using a homemade antibody previously characterized [10]. The immunoprecipitates were separated by Tricine-SDS polyacrylamide-gel-electrophoresis (PAGE) to resolve peptides below 10-15 kDa and were visualized using autoradiography. In addition to full-length PGRN, a band of approximately 10 kDa, corresponding to the expected size of granulin peptides, was present in wild type (WT) mouse microglia but absent in *Grn* ^−/−^ microglia (Fig. 1a), indicating that these were peptides derived from PGRN. Although PGRN has been shown to be cleaved by elastase and MMPs extracellularly [14, 15], we failed to detect any significant processed PGRN products in the secreted fraction (Fig. 1a), suggesting that PGRN is primarily processed intracellularly in microglia.

Previously, we reported an interaction between PGRN and PSAP [10]. However, PGRN does not bind to processed saposin peptides [10, 16]. While, based on the autoradiography results alone, we can not rule out that there might be other peptides interacting with PGRN, most likely the peptides that we visualized are PGRN-derived. To confirm this, we attempted to detect these peptides via Western blotting. A clear band of approximately 10 kDa was detected in lysates from the wild type mouse embryonic fibroblasts (MEFs) but was absent from *Grn* ^−/−^ fibroblasts using commercial polyclonal anti-mouse PGRN antibodies (Fig. 1b). This band was also detected in the brain, liver, spleen, kidney, lung and heart lysates from wild type mice (Fig. 1c, d), but was absent in lysates derived from *Grn* ^−/−^ tissues (Fig. 1d). These data further support the existence of intracellular, PGRN-derived peptides in multiple cell and tissue types. Because PGRN haploinsufficiency causes FTLD, we also tested whether the rate of PGRN processing is altered in *Grn* ^+/−^ versus WT spleen. The amount of both PGRN and granulin peptides is reduced in *Grn* ^+/−^ samples and there is no statistically significant difference in PGRN processing between WT and *Grn* ^+/−^ (Fig. 1e).

### PGRN processing is lysosome-dependent

**Fig. 2:**
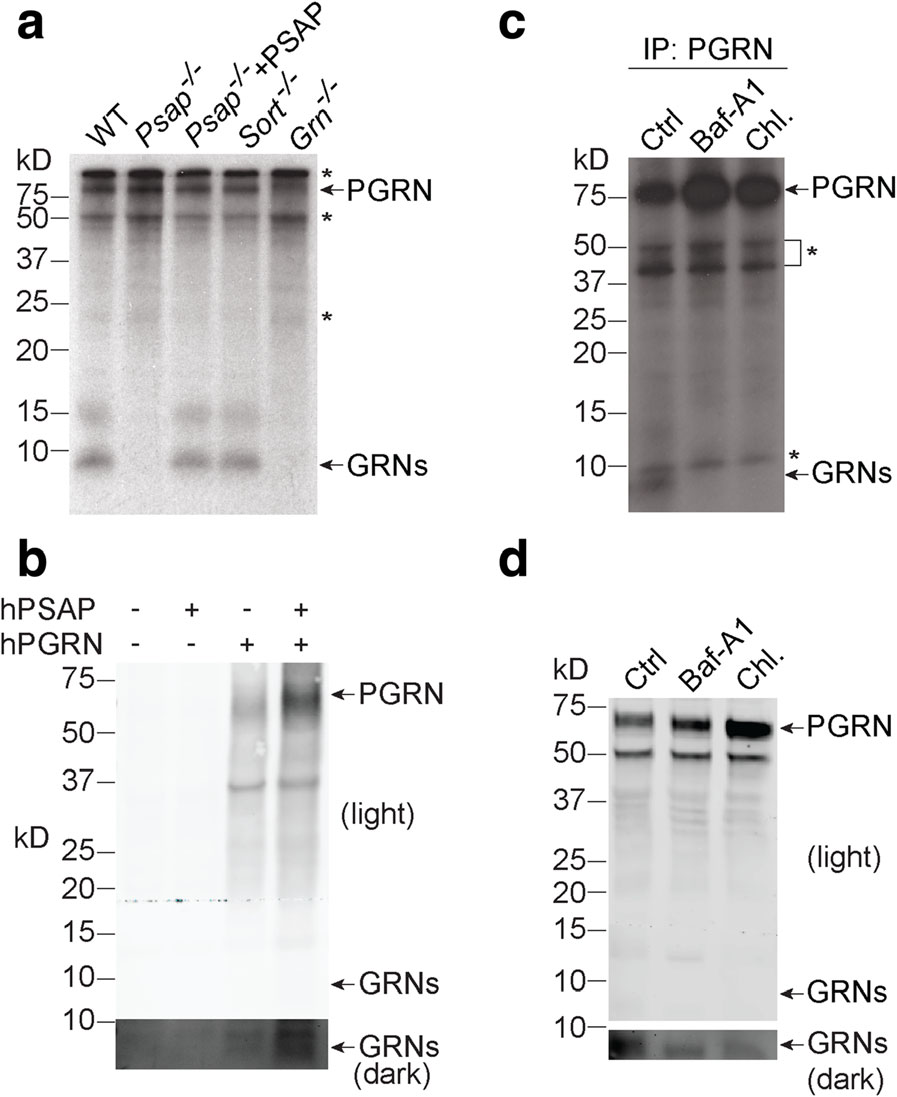
PGRN processing is lysosome-dependent. **a** Primary *Sort* ^*−/−*^, *Grn* ^*−/−*^, and *Psap* ^*−/−*^ MEF cells, and *Psap* ^*−/−*^ MEF cells infected with *PSAP* lentivirus were labeled with [^35^S]-methionine and [^35^S]-cysteine for 24 h. Equal amounts of cell lysate were immunoprecipitated with a homemade rabbit anti-PGRN antibody and separated by 16% Tricine-SDS PAGE. The PGRN and PGRN-derived peptide (GRNs) signals were visualized by autoradiography. * indicates non-specific bands. **b** PGRN delivered from the extracellular space is processed in primary cortical neurons (DIV12). Primary cortical neurons were treated with either human PGRN (hPGRN, 1 μg/ml) alone or together with recombinant human PSAP (hPSAP, 1 μg/ml) as indicated for 16 h. The cells were harvested and proteins were separated on a 4–12% Bis-Tris gel, then blotted with goat anti-human PGRN antibodies. **c** Intracellular processing of PGRN is dependent on lysosomal activity. Primary MEF cells were labeled with [^35^S]-methionine and [^35^S]-cysteine and treated with different lysosomal inhibitors: 50 nM bafilomycin or 15 mM ammonium chloride + 100 μM chloroquine for 16 h. The cell lysates were immunoprecipitated with rabbit anti-PGRN antibodies and separated by 16% Tricine-SDS PAGE. PGRN and PGRN-derived peptides were visualized by autoradiography. * indicates non-specific bands. **d** Primary MEF cells were treated with different lysosomal inhibitors, as above. The cell lysates were separated on a Bis-Tris gel, then blotted with sheep anti-mouse PGRN antibodies

It was previously shown that PGRN is localized to lysosomes within the cell [9, 10]. Although sortilin is the canonical lysosomal trafficking receptor for PGRN, we have recently shown that PSAP, but not sortilin, is required for PGRN lysosomal trafficking in fibroblasts, which express only negligible levels of sortilin [10]. To determine whether lysosomal trafficking is required for PGRN processing, we assessed PGRN processing in WT and *Psap* ^−/−^ fibroblasts. PSAP ablation totally abolished PGRN processing, which could be rescued by expression of PSAP with a viral vector (Fig. 2a). Furthermore, PGRN processing was normal in fibroblasts in which sortilin had been deleted [10] (Fig. 2a). Taken together, these data suggest that lysosomal trafficking is required for PGRN processing.

Because PGRN can also be delivered to lysosomes from the extracellular space [9, 10], we wanted to determine whether extracellular-derived, endocytosed PGRN can also be processed. To assess this, we treated primary *Grn* ^−/−^ cortical neurons with purified recombinant human PGRN. A band of approximately 10 kDa, corresponding to the size of granulin peptides, was detected when extracellular PGRN was added (Fig. 2b). Furthermore, PGRN uptake and lysosomal delivery is known to be enhanced by PSAP [10]. Consistent with this, the presence of recombinant PSAP greatly facilitated neuronal uptake of full-length PGRN and also increased the levels of processed granulin peptides (Fig.2b).

To test the direct role of the lysosome in PGRN processing, we treated MEFs with lysosomal inhibitors known to interfere with lysosomal acidification. Either bafilomycin A1, alone, or chloroquine with ammonium chloride were used. Both bafilomycin A1 and chloroquine/ammonium chloride treatment led to the reduction of the 10 kDa granulin peptide bands and increased levels of full-length PGRN with both radiolabeling and Western blot analysis (Fig. 2c, d), supporting that proper lysosomal function is required for intracellular PGRN processing.

### PGRN processing is dependent on cathepsins

**Fig. 3:**
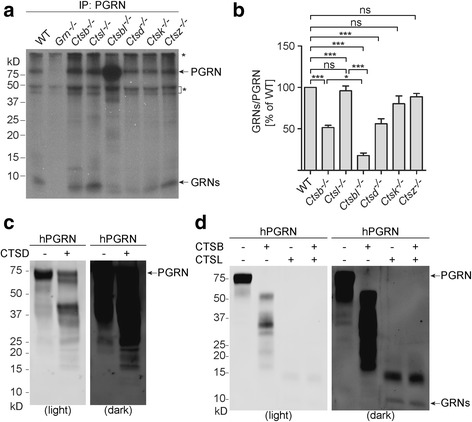
PGRN processing by cathepsins. **a** Cathepsin- and PGRN-deficient MEF cells were labeled with [^35^S]-methionine and [^35^S]-cysteine for 24 h and the cell lysates were then immunoprecipitated with rabbit anti-PGRN antibodies and separated by 16% Tricine-SDS PAGE. PGRN and PGRN-derived peptides were visualized by autography. * indicates non-specific bands. **b** Quantification of PGRN and PGRN-derived peptides in (a). 10 kDa PGRN-derived peptides were normalized with full-length PGRN signals in each group. Data is presented as means ± s.e.m. *n* = 3, * P,0.05; ***P < 0.001, ns, not significant, one-way ANOVA, Tukey’s Multiple Comparison Test. **c** Recombinant cathepsin D was incubated with recombinant human PGRN in acidic buffer for 16 h. Proteins were separated on a Bis-Tris gel and blotted with goat anti-human PGRN antibodies. **d** Recombinant cathepsin B and L were incubated with recombinant human PGRN, as indicated, in acidic buffer for 4 h. Proteins were separated on a Bis-Tris gel and blotted with goat anti-human PGRN antibodies

Because cathepsins are the main proteases in the lysosome [17, 18], we predicted that one or more could be involved in PGRN processing. To determine the role of several well-studied cathepsins in PGRN processing, we tested PGRN processing in fibroblasts deficient in either cathepsin B (Ctsb), cathepsin L (Ctsl), cathepsin D (Ctsd), cathepsin K (Ctsk), cathepsin Z (Ctsz), or deficient in both cathepsin B (Ctsb) and cathepsin L (Ctsl), which were derived from available knockout mice. Deletion of cathepsin L, K or Z had no effect on PGRN processing, while ablation of either cathepsin B or D resulted in ~50% reduction in the ratio of processed PGRN peptides versus full-length PGRN (Fig. 3a and b). Interestingly, ablation of both cathepsin B and L resulted in a much greater decrease in PGRN processing than cathepsin B deletion alone (Fig. 3a, b), suggesting that cathepsins B and L might play redundant roles in PGRN processing, which is consistent with reported redundancy between these enzymes [19]. To determine the direct roles of cathepsins B, D and L in PGRN processing, we tested the ability of recombinant cathepsins to cleave recombinant PGRN in vitro. While cathepsin B and D were capable of cleaving PGRN to a minor degree, incubation of recombinant PGRN with cathepsin L led to the generation of bands of approximately 10 kDa, corresponding to the size of granulin peptides (Fig. 3c, d). This is consistent with another study published while our manuscript was under revision [20], in which they confirmed that cathepsin L cleaves PGRN in the linker regions between granulin peptides using mass spectrometry. These data suggest that cathepsins are the key lysosomal enzymes involved in intracellular PGRN processing.

## Discussion

Many lysosomal proteins are known to undergo processing in the acidic environment. One example is PSAP, which is known to be processed into saposin peptides (A, B, C, D) in the lysosome [11–13]. In this manuscript, we showed that PGRN, the precursor of granulin peptides, is processed intracellularly in a lysosome-dependent manner and that multiple cathepsins are likely to be involved in this processing. While our in vitro analysis demonstrated that cathepsin L is very potent in processing PGRN to granulin peptides (Fig. 3d), cathepsin L - deficient MEFs do not show obvious defects in PGRN processing (Fig. 3a, b), suggesting there is another protease playing a role redundant to cathepsin L in vivo. MEFs deficient in both cathepsin B and L have minimal ability to process PGRN (Fig. 3a, b) despite cathepsin B being minorly active towards PGRN in vitro (Fig. 3d), indicating that cathepsin B is not the enzyme directly processing PGRN in cathepsin L - deficient MEFs. However, lysosomal enzymes are often activated by the action of other enzymes, especially in the case of cathepsins. Thus it is possible that an unidentified protease that is activated by cathepsin B is responsible for processing PGRN in the absence of cathepsin L.

Our data also showed that PGRN is processed to the ~ 10 kDa peptides in multiple cell types and tissues and both mouse and human PGRN are processed in a similar manner. This suggests that lysosomal PGRN processing is a general phenomenon present in all cell types and conserved during evolution. While it is likely that these ~ 10 kDa peptides are a mixture of granulin peptides, the exact sequences of these peptides need to be further analyzed. Future studies using mass spectrometry and the development of tools and reagents to characterize individual granulin peptides will allow a better understanding of PGRN processing.

Recently, PGRN was shown to physically interact with cathepsin D and regulate its activity and multiple granulins are involved in this interaction [21, 22]. One intriguing possibility is that granulins modulate cathepsin activities in the lysosome. Different granulins might also interact with different proteins in the lysosome in a manner similar to how saposins activate different enzymes in the glycosphingolipid degradation pathway. Future endeavors to identify lysosomal proteins interacting with these granulin peptides will help us to obtain a better understanding of their functions in the lysosome.

## Conclusion

Our data support that PGRN is processed in a lysosome-dependent manner and cathepsin L cleaves PGRN in vitro. Further studies on the interacting partners of these processed peptides will provide a better understanding of PGRN function in the lysosome.

## Methods

### Pharmacological reagents and antibodies

The following antibodies were used in this study: goat anti-human PGRN (1:1000 for Western blot) and sheep anti-mouse PGRN (1:300 for brain lysate Western blot, 1:1000 for other Western blots,) from R&D systems. Recombinant cathepsin D and L proteins were from R&D systems. Bafilomycin A1, ammonium chloride and chloroquine were from Sigma.

### Expression constructs

Human CTSB and CTSD cDNA in the pDONR223 vector were obtained from the ORFeome Collection (kind gifts from Dr. Haiyuan Yu). CTSB-myc-His and CTSD-myc-His constructs were generated using a gateway reaction with pDONR223-CTSB/CTSD and a modified pcDNA3.1/myc-His A vector (Invitrogen), engineered with a gateway cassette.

### Cell culture, DNA transfection, protein purification, and PSAP lentivirus production and infection

HEK293T were maintained in Dulbecco’s Modified Eagle’s medium (Cellgro) supplemented with 10% fetal bovine serum (Gibco) and 1% Penicillin–Streptomycin (Invitrogen) in a humidified incubator at 37 °C and 5% CO_2_. Cells were transiently transfected with polyethylenimine as described [23]. Conditioned medium from cells transfected with the Cathepsin B-myc-His construct was collected 4 days after transfection and incubated with cobalt beads. After extensive washing, recombinant Cathepsin B was eluted with imidazole and dialyzed to PBS buffer. Recombinant human PGRN was purified from the conditioned medium of transfected HEK293T cells as described [10]. Primary microglia, cortical neurons, and fibroblasts were cultured as described [10]. Primary cathepsin KO fibroblasts were derived from *ctsd−/−*[24], *ctsb−/−* [25], *ctsl−/−* [26], *ctsb−/− ctsl−/−* [19], *ctsk−/−* [27] and *ctsz−/−* [28] mice. PSAP lentiviruses were generated from HEK293T cells and then used to infect *Psap* ^−/−^ fibroblasts as described [10].

### Metabolic labeling and PGRN processing assay

To obtain [^35^S]-labeled PGRN, culture medium was replaced with methionine- and cysteine-free DMEM with 10% dialyzed FBS for 2 h before the addition of [^35^S]-labeled methionine and cysteine. After 24 h incubation, cells were lysed with lysis buffer (50 mM Tris, pH 7.3, 150 mM NaCl, 1% Triton X-100, and 0.1% deoxycholate with protease inhibitors). After immunoprecipitation with homemade rabbit anti-PGRN antibodies [10], the IP products were separated by 16% Tricine SDS-PAGE. Fixation solution (10% methanol and 10% acetic acid) was added, the gels were subsequently saturated with amplification solution (1 M sodium salicylate, 10% glycerol), and the autoradiographs of dried gels were obtained on X-ray film at −80 °C.

### In vitro cleavage of PGRN by cathepsins

1 μg of recombinant human PGRN and 0.25 μg of recombinant cathepsin B, D, or L, or PBS control were combined and pre-incubated for 0.5 h on ice. 3× assay buffer (150 mM NaOAc pH 5.3, 12 mM EDTA, 24 mM DTT for cathepsin B and L; 300 mM NaOAc, 0.6 M NaCl, pH 3.5 for cathepsin D) was added and brought to 1× by the addition of H_2_O to a final volume of 15 μl. The reactions were kept at 37 °C for 4 h (cathepsin B and L) or 16 h (cathepsin D) and the reaction stopped by the addition of 2× Laemmli sample buffer. Proteins were separated on a 4–12% Bis-Tris gel and visualized using Western blotting with goat anti-human PGRN antibodies.

### Western blot analysis

Samples were separated by 4–12% Bis-Tris PAGE (Invitrogen) and transferred to 0.2 μm nitrocellulose. Western blot analysis was performed using anti-PGRN antibodies as described [10].

## Abbreviations

Cts: Cathepsin
FTLD: Frontotemporal lobar degeneration
MEF: Mouse embryonic fibroblast
NCL: Neuronal ceroid lipofuscinosis
PGRN: Progranulin

## References

[1] Ahmed Z, Mackenzie IR, Hutton ML, Dickson DW (2007). Progranulin in frontotemporal lobar degeneration and neuroinflammation. J Neuroinflammation.

[2] Cenik B, Sephton CF, Kutluk Cenik B, Herz J, Yu G (2012). Progranulin: a proteolytically processed protein at the crossroads of inflammation and neurodegeneration. J Biol Chem.

[3] Bateman A, Bennett HP (2009). The granulin gene family: from cancer to dementia. BioEssays.

[4] Nicholson AM, Gass J, Petrucelli L, Rademakers R (2012). Progranulin axis and recent developments in frontotemporal lobar degeneration. Alzheimers Res Ther.

[5] Neary D, Snowden JS, Gustafson L, Passant U, Stuss D, Black S (1998). Frontotemporal lobar degeneration: a consensus on clinical diagnostic criteria. Neurology.

[6] Smith KR, Damiano J, Franceschetti S, Carpenter S, Canafoglia L, Morbin M (2012). Strikingly different clinicopathological phenotypes determined by progranulin-mutation dosage. Am J Hum Genet.

[7] Almeida MR, Macario MC, Ramos L, Baldeiras I, Ribeiro MH, Santana I. Portuguese family with the co-occurrence of frontotemporal lobar degeneration and neuronal ceroid lipofuscinosis phenotypes due to progranulin gene mutation. Neurobiol Aging. 2016. doi: 10.1016/j.neurobiolaging.2016.02.019. 10.1016/j.neurobiolaging.2016.02.019 27021778

[8] Belcastro V, Siciliano V, Gregoretti F, Mithbaokar P, Dharmalingam G, Berlingieri S (2011). Transcriptional gene network inference from a massive dataset elucidates transcriptome organization and gene function. Nucleic Acids Res.

[9] Hu F, Padukkavidana T, Vaegter CB, Brady OA, Zheng Y, Mackenzie IR (2010). Sortilin-mediated endocytosis determines levels of the frontotemporal dementia protein, progranulin. Neuron.

[10] Zhou X, Sun L, Bastos de Oliveira F, Qi X, Brown WJ, Smolka MB (2015). Prosaposin facilitates sortilin-independent lysosomal trafficking of progranulin. J Cell Biol.

[11] O'Brien JS, Kishimoto Y (1991). Saposin proteins: structure, function, and role in human lysosomal storage disorders. FASEB J.

[12] Qi X, Grabowski GA (2001). Molecular and cell biology of acid beta-glucosidase and prosaposin. Prog Nucleic Acid Res Mol Biol.

[13] Matsuda J, Yoneshige A, Suzuki K (2007). The function of sphingolipids in the nervous system: lessons learnt from mouse models of specific sphingolipid activator protein deficiencies. J Neurochem.

[14] Zhu J, Nathan C, Jin W, Sim D, Ashcroft GS, Wahl SM (2002). Conversion of proepithelin to epithelins: roles of SLPI and elastase in host defense and wound repair. Cell.

[15] Suh HS, Choi N, Tarassishin L, Lee SC (2012). Regulation of progranulin expression in human microglia and proteolysis of progranulin by matrix metalloproteinase-12 (MMP-12). PLoS One.

[16] Zhou X, Sullivan PM, Sun L, Hu F. The interaction between progranulin and prosaposin is mediated by granulins and the linker region between saposin B and C. J Neurochem. 2017; 10.1111/jnc.14110. 10.1111/jnc.14110 PMC5630500 28640985

[17] Stoka V, Turk V, Turk B (2016). Lysosomal cathepsins and their regulation in aging and neurodegeneration. Ageing Res Rev.

[18] Ketterer S, Gomez-Auli A, Hillebrand LE, Petrera A, Ketscher A, Reinheckel T. Inherited diseases caused by mutations in cathepsin protease genes. FEBS J. 2016;doi: 10.1111/febs.13980. 10.1111/febs.13980 27926992

[19] Sevenich L, Pennacchio LA, Peters C, Reinheckel T (2006). Human cathepsin L rescues the neurodegeneration and lethality in cathepsin B/L double-deficient mice. Biol Chem.

[20] Lee CW, Stankowski JN, Chew J, Cook CN, Lam YW, Almeida S (2017). The lysosomal protein cathepsin L is a progranulin protease. Mol Neurodegener.

[21] Beel S, Moisse M, Damme M, De Muynck L, Robberecht W, Van Den Bosch L, et al. Progranulin functions as a cathepsin D chaperone to stimulate axonal outgrowth in vivo. Hum Mol Genet. 2017; 10.1093/hmg/ddx162. 10.1093/hmg/ddx162 PMC5886064 28453791

[22] Zhou X, Paushter DH, Feng T, Pardon CM, Mendoza CS, Hu F. Regulation of cathepsin D activity by the FTLD protein progranulin. Acta Neuropathol. 2017; 10.1007/s00401-017-1719-5. 10.1007/s00401-017-1719-5 PMC5568051 28493053

[23] Vancha AR, Govindaraju S, Parsa KV, Jasti M, Gonzalez-Garcia M, Ballestero RP (2004). Use of polyethyleneimine polymer in cell culture as attachment factor and lipofection enhancer. BMC Biotechnol.

[24] Saftig P, Hetman M, Schmahl W, Weber K, Heine L, Mossmann H (1995). Mice deficient for the lysosomal proteinase cathepsin D exhibit progressive atrophy of the intestinal mucosa and profound destruction of lymphoid cells. EMBO J.

[25] Halangk W, Lerch MM, Brandt-Nedelev B, Roth W, Ruthenbuerger M, Reinheckel T (2000). Role of cathepsin B in intracellular trypsinogen activation and the onset of acute pancreatitis. J Clin Invest.

[26] Roth W, Deussing J, Botchkarev VA, Pauly-Evers M, Saftig P, Hafner A (2000). Cathepsin L deficiency as molecular defect of furless: hyperproliferation of keratinocytes and pertubation of hair follicle cycling. FASEB J.

[27] Saftig P, Hunziker E, Wehmeyer O, Jones S, Boyde A, Rommerskirch W (1998). Impaired osteoclastic bone resorption leads to osteopetrosis in cathepsin-K-deficient mice. Proc Natl Acad Sci U S A.

[28] Sevenich L, Schurigt U, Sachse K, Gajda M, Werner F, Muller S (2010). Synergistic antitumor effects of combined cathepsin B and cathepsin Z deficiencies on breast cancer progression and metastasis in mice. Proc Natl Acad Sci U S A.

